# Two new species of Pseudolimnophila (Pseudolimnophila) Alexander, 1919 (Diptera, Limoniidae) from northern China

**DOI:** 10.3897/zookeys.1273.174825

**Published:** 2026-03-17

**Authors:** Le Shui, Chenbin Wang, Yanjing Zhang, Fangzhou Ma, Ping Wang, Bing Zhang, Ding Yang

**Affiliations:** 1 Nanjing Institute of Environmental Sciences, Ministry of Ecology and Environment, Key Laboratory of Biodiversity and Biosafety, Nanjing 210042, China School of Chemistry and Chemical Engineering, Xianyang Normal University Xianyang China; 2 School of Chemistry and Chemical Engineering, Xianyang Normal University, Xianyang 712000, China College of Plant Protection, China Agricultural University Beijing China; 3 The Youth Innovation Team of Shaanxi Universities, Xianyang 712000, China Nanjing Institute of Environmental Sciences, Ministry of Ecology and Environment Nanjing China; 4 Engineering Research Center of Loess Plateau Authentic Medicinal Herbs, Universities of Shaanxi Province, Xianyang 712000, China Engineering Research Center of Loess Plateau Authentic Medicinal Herbs, Universities of Shaanxi Province Xianyang China; 5 Department of Entomology, College of Plant Protection, China Agricultural University, Beijing 100193, China The Youth Innovation Team of Shaanxi Universities Xianyang China

**Keywords:** Crane flies, Limnophilinae, *
Pseudolimnophila
*, taxonomy

## Abstract

Only one species of the subgenus Pseudolimnophila (Pseudolimnophila) Alexander, 1919 was previously known from northern China. Herein, two new species from northern China are described: Pseudolimnophila (Pseudolimnophila) chanbaensis Shui, Zhang B. & Yang, **sp. nov**. and P. (P.) shanxiensis Shui, Zhang B. & Yang, **sp. nov**. Additionally, P. (P.) inconcussa (Alexander, 1913) is reported for the first time from Shaanxi Province and redscribed. A key to the species of the subgenus *Pseudolimnophila* occurring in China is provided.

## Introduction

The morphologically diverse genus *Pseudolimnophila* Alexander, 1919 is composed of two subgenera, *Calolimnophila* Alexander, 1921 with 13 species in the Afrotropics, and *Pseudolimnophila* with 64 species worldwide except Australasia (Oosterbroek 2025).

The subgenus Pseudolimnophila (Pseudolimnophila) Alexander, 1919 belongs to the family Limoniidae and is distributed worldwide, with 64 known species and subspecies. Among these, seven taxa occur in the Palaearctic region, six in the Nearctic region, five in the Neotropical region, 29 in the Oriental region, and 19 in the Afrotropical region (Oosterbroek 2025). It is characterized by the following features: small to medium-sized body; antennae with 14 flagellomeres; pronotum narrow, with margins produced anteriorly into lateral lobes; wings often unpatterned or variously darkened; cell *dm* present; three or four branches of media vein (*M*) reaching the margin; clasper of gonostylus apically narrowed into a long, curved spine; lobe of gonostylus short and stout, bearing setae; gonocoxite moderately stubby or elongate-cylindrical; interbase usually two branches; aedeagus usually varying in length and directed dorsally (Alexander 1948; Alexander and Byers 1981; Reusch and Oosterbroek 1997; Podenas et al. 2006; Ribeiro 2008; de Jong 2017).

To date, only seven species of the subgenus *Pseudolimnophila* have been known to occur in China (Ren et al. 2020; Oosterbroek 2025): P. (P.) inconcussa (Alexander, 1913) from the Eastern Palaearctic and Oriental region; P. (P.) brunneinota Alexander, 1933 from the Eastern Palaearctic region; and P. (P.) chikurina Alexander, 1930, P. (P.) concussa Alexander, 1936, P. (P.) descripta Alexander, 1928, P. (P.) projecta Alexander, 1937, and P. (P.) seticostata Alexander, 1936 from the Oriental region. To enrich the understanding of the species composition of Limnophilinae, we conducted a scientific survey of crane flies in northern China from 2022 to 2025. Currently, three species of the subgenus *Pseudolimnophila*—including two new species—are added to the fauna of northern China: P. (P.) inconcussa is reported for the first time from Shaanxi Province; P. (P.) chanbaensis Shui, Zhang B. & Yang, sp. nov. is described and illustrated from Shaanxi Province; and P. (P.) shanxiensis Shui, Zhang B. & Yang, sp. nov. is described and illustrated from Shanxi Province. A key to the species of the subgenus *Pseudolimnophila* from China is provided.

## Materials and methods

The specimens were examined and illustrated using a ZEISS Stemi 2000-c stereomicroscope. Details of their colouration were checked in specimens immersed in 75% ethyl alcohol. Male genitalia were prepared by macerating the apical portion of the abdomen in cold 10% NaOH for 12–15 h. After examination, the genitalia were transferred to fresh glycerol (C_3_H_8_O_3_) and stored in a microvial pinned beneath the respective specimen. The studied specimens, collected from Shaanxi and Shanxi provinces, are deposited in the Entomological Museum of China Agricultural University (**CAU**), Beijing, China.

Specimen dissection and morphological examination: adult specimens were dissected to expose terminalia for taxonomic analysis. For male specimens, genitalia were carefully dissected under a stereomicroscope to ensure the integrity of diagnostic structures. Line drawings of male terminalia were prepared based on these dissected materials following microscopic examination. These drawings represent the key diagnostic structures that were utilized for species delimitation in the present study.

Note on female genitalia examination: a limitation of this study is the lack of examination of internal female genital structures, despite high-resolution images of external genitalia’s lateral traits and oviposition processes. Genital characters have notable diagnostic value for taxonomy; their analysis can provide key data for species delimitation in this genus. Future revisions should prioritize dissecting, examining, and documenting these internal structures to improve classification accuracy.

The terminology used for the wing veins follows the interpretation of de Jong (2017). The terminology for the male terminalia follows that of Ribeiro (2006, 2008). The following abbreviations are used in the figures: **8s** = eighth sternite; **8t** = eighth tergite; **9s** = ninth sternite; **9t** = ninth tergite; **10s** = tenth sternite; **10t** = tenth tergite; **goncx** = gonocoxite; **cgonst** = clasper of gonostylus; **lgonst** = lobe of gonostylus; **interb** = interbase; **aed** = aedeagus; **ce** = cercus; **hy** = hypogynial valve.

## Taxonomy

Species list of the subgenus *Pseudolimnophila* recorded in China:

1. P. (P.) brunneinota Alexander, 1933: Sichuan, Xizang
2. P. (P.) chanbaensis Shui, Zhang B. & Yang, sp. nov.: Shaanxi
3. P. (P.) chikurina Alexander, 1930: Taiwan
4. P. (P.) concussa Alexander, 1936: Hainan
5. P. (P.) descripta Alexander, 1928: Taiwan
6. P. (P.) inconcussa (Alexander, 1913): Taiwan, Shaanxi, Guangdong, Guangxi
7. P. (P.) projecta Alexander, 1937: Guangdong
8. P. (P.) seticostata Alexander, 1936: Hainan
9. P. (P.) shanxiensis Shui, Zhang B. & Yang, sp. nov.: Shanxi

### Key to species of the subgenus *Pseudolimnophila* from China (adult)

**Table:** 

1	*M* with four branches (Figs 3A, 5A, 7A)	**2**
–	*M* with three branches (Alexander 1936: pl. 1, fig. 10; Alexander 1928: pl. 1, fig. 8; Alexander 1937: pl. 1, fig. 14)	**7**
2	*R_2_* placed after fork of *R_3+4_* (Fig. 7A)	**3**
–	*R_2_* not placed after fork of *R_3+4_* (Figs 3A, 5A)	**5**
3	*M_1_* longer, approximately 2 times as long as *M_1+2_* (Fig. 7A)	**P. (P.) shanxiensis**
–	*M_1_* shorter, approximately as long as *M_1+2_* (Alexander 1933: pl. 1, fig. 6; Alexander 1930: pl. 1, fig. 8)	**4**
4	Pleura yellowish (Alexander 1933: 141)	**P. (P.) brunneinota**
–	Pleura brownish grey (Alexander 1930: 70)	**P. (P.) chikurina**
5	*R_2_* placed before fork of *R_3+4_* (Fig. 3A)	**P. (P.) chanbaensis**
–	*R_2_* placed at fork of *R_3+4_* (Fig. 5A; Alexander 1936: pl. 1, fig. 9)	**6**
6	Male interbase with a low obtuse flange at near midlength (Alexander 1936: 127)	**P. (P.) concussa**
–	Male interbase without obtuse flange (Fig. 5C–E)	**P. (P.) inconcussa**
7	Cell *dm* large, approximately 1/2 as long as *M_1+2_* (Alexander 1937: pl. 1, fig. 14)	**P. (P.) projecta**
–	Cell *dm* small, approximately 1/3 as long as *M_1+2_* (Alexander 1936: pl. 1, fig. 10; Alexander 1928: pl. 1, fig. 8)	**8**
8	Prescutum brown with three scarcely evident darker brown stripes (Alexander 1928: 475)	**P. (P.) descripta**
–	Prescutum almost uniformly dark brown without darker brown stripes (Alexander 1936: 128)	**P. (P.) seticostata**

#### Pseudolimnophila (Pseudolimnophila) chanbaensis

Taxon classificationAnimaliaDipteraLimoniidae

Shui, Zhang B. & Yang sp. nov.

A3F843CF-C234-5F43-83C4-70AB1097EA35

https://zoobank.org/54F37BA3-0BF9-4313-9E85-773157E89857

Figs 1, 2, 3

##### Type material.

**Holotype**: • male (CAU), China: Shaanxi, Xi’an Chanba National Wetland Park, 34.42N, 108.99E, 368 m, 2022.IV.12, Bing Zhang, CAU: Limn: 129072. **Paratypes**: • 8 males, 12 females (CAU), China: Shaanxi, Xi’an Chanba National Wetland Park, 34.42N, 108.99E, 368 m, 2022.IV.12, Bing Zhang, CAU: Limn: 129073, CAU: Limn: 129074, CAU: Limn: 129075, CAU: Limn: 129076, CAU: Limn: 129077, CAU: Limn: 129078, CAU: Limn: 129079, CAU: Limn: 129080, CAU: Limn: 129081, CAU: Limn: 129082, CAU: Limn: 129083, CAU: Limn: 129084, CAU: Limn: 129085, CAU: Limn: 129086, CAU: Limn: 129087, CAU: Limn: 129088, CAU: Limn: 129089, CAU: Limn: 129090, CAU: Limn: 129091, CAU: Limn: 129092; • 1 female (CAU), China: Shaanxi, Xianyang, Qinhan New City Weihe Wetland Park, 34.40N, 108.88E, 378 m, 2025.IV.11, Bing Zhang, CAU: Limn: 129093; • 4 females (CAU), China: Shaanxi, Xi’an Chanba National Wetland Park, 34.42N, 108.99E, 368 m, 2025.IV.25, Bing Zhang, Le Shui and Chengzhang Li, CAU: Limn: 129094, CAU: Limn: 129095, CAU: Limn: 129096, CAU: Limn: 129097.

**Figure 1.**
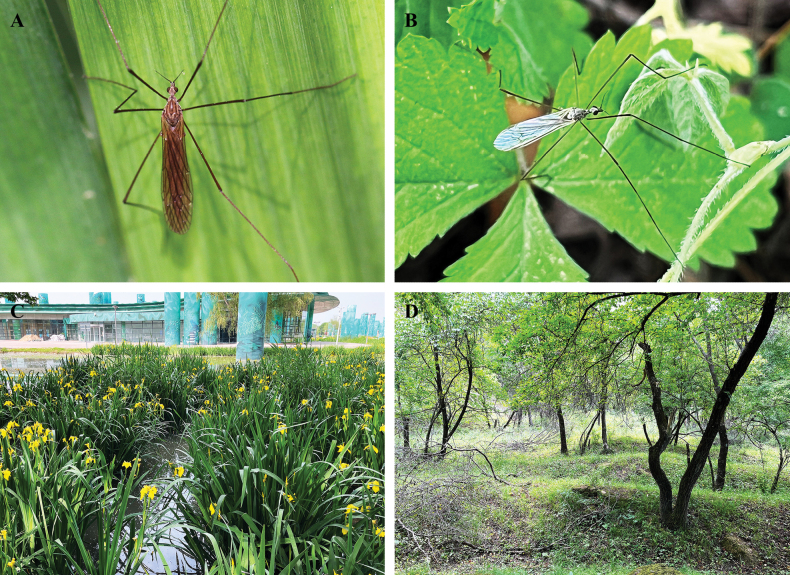
**A**, **C**. Habitus and habitat of Pseudolimnophila (P.) chanbaensis Shui, Zhang B. & Yang, sp. nov.; **B, D**. Habitus and habitat of P. (P.) inconcussa.

##### Diagnosis.

Prescutum brownish yellow with four dark brown stripes; mesopleura brownish yellow with pale grey pruinosity; anepisternum brownish black; katepisternum brownish yellow with pale grey pruinosity, posterior margin dark brown; *R_2_* not oblique, placed before fork of *R_3+4_*, approximately as long as 1/2 of *R_3+4_*; *M_1_* approximately as long as *M_1+2_*; abdominal sternites 1–8 yellow; interbase with two branches, outer-lateral branch short and small with spine-like apex, inner branch as long as aedeagus, slightly wider at base, middle to posterior part slender and rod-shaped; aedeagus elongated and rod-shaped, with a thick base that gradually tapers toward the tip.

##### Description.

**Male** (*n* = 9): body length 5.8**–**6.4 mm, wing length 6.8**–**7.2 mm, antenna length 1.1–1.2 mm.

**Figure 2.**
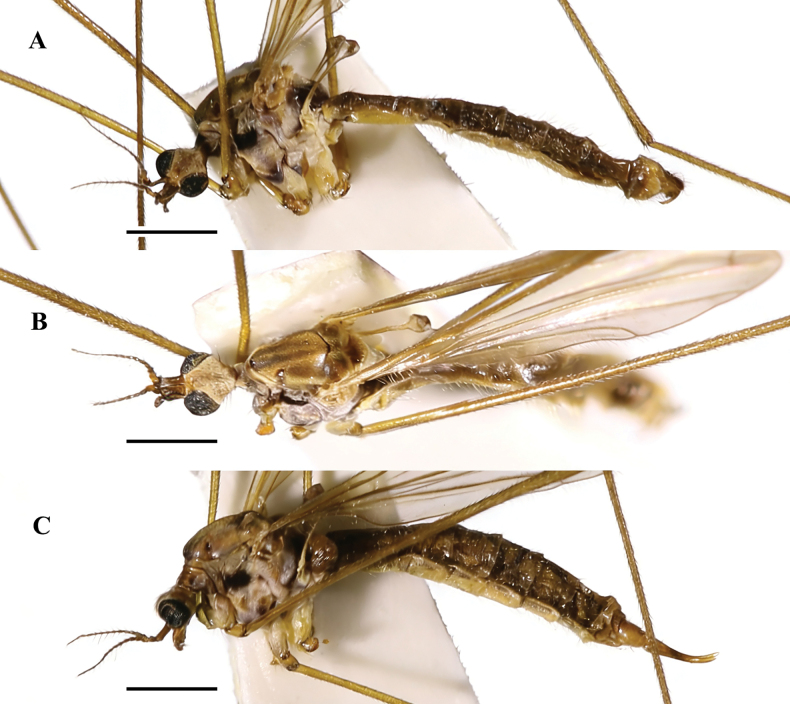
Pseudolimnophila (P.) chanbaensis Shui, Zhang B. & Yang, sp. nov. **A**. Male habitus, lateral view; **B**. Male habitus, dorsal view; **C**. Female habitus, lateral view. Scale bars: 1.0 mm (**A**–**C**).

***Head*** (Fig. 2A, B) brownish with pale grey pruinosity. Vertex brownish yellow with long setae; scape and pedicel dark yellow; flagellomeres dark brown, cylindrical with longer verticils; rostrum and palpus greyish black with black setae.

**Figure 3.**
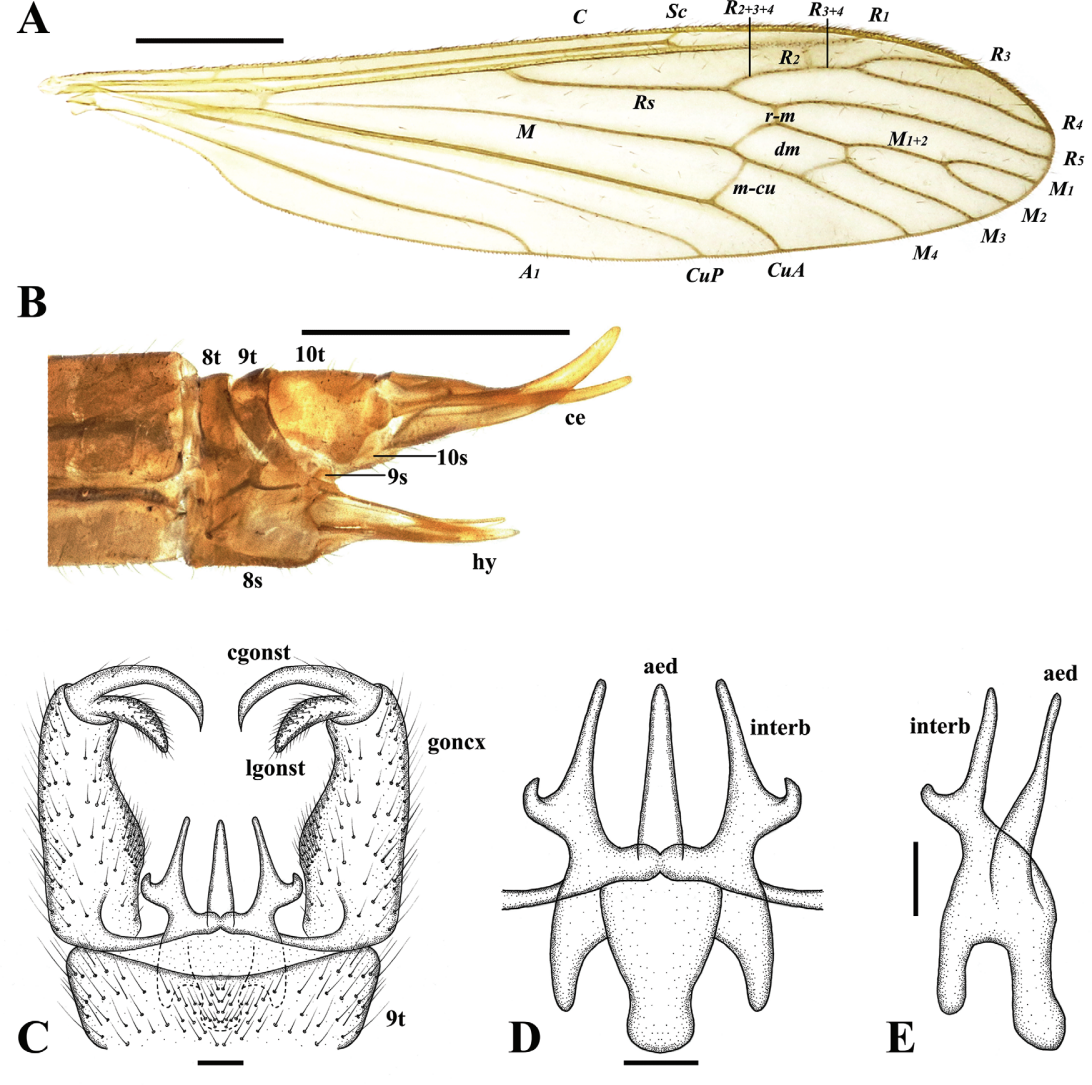
Pseudolimnophila (P.) chanbaensis Shui, Zhang B. & Yang, sp. nov. **A**. Male wing; **B**. Ovipositor, lateral view; **C**. Terminalia, dorsal view; **D**. Aedeagal complex, dorsal view; **E**. Aedeagal complex, lateral view. Scale bars: 1.0 mm (**A**, **B**); 0.1 mm (**C**–**E**).

***Thorax*** (Fig. 2A, B) brown with pale grey pruinosity. Pronotum brownish yellow; prescutum brownish yellow with four dark brown stripes, middle two dark brown stripes longer, near pronotum darker; prescutal suture brownish black; scutum dark brown; scutellum brownish yellow; mediotergite brownish black; laterotergite brownish yellow with grey pruinosity; anepisternum brownish black; katepisternum brownish yellow with pale grey pruinosity, posterior margin dark brown; anepimeron brownish yellow with pale grey pruinosity; katepimeron brownish yellow with pale grey pruinosity, posterior margin dark brown; metapleuron light yellowish with pale pruinosity. Legs: coxa light yellowish with long brownish setae; trochanter brownish yellow; femur brownish yellow with brown setae, apically dark brown; tibia and tarsus brownish yellow with brown setae, apically darker. Wing (Fig. 3A) pale brownish yellow hyaline, anal cell paler; pterostigma pale brown. Venation: *R_2_* not oblique, placed before fork of *R_3+4_*, approximately as long as 1/2 of *R_3+4_*; *M_1_* approximately as long as *M_1+2_*; *m-cu* oblique, near the 1/4 base of cell *dm*. Halter (Fig. 2A, B) length approximately 1.3 mm, basal 1/4 of stem bright yellow with pale pruinosity, remainder of stem and knob greyish brown.

***Abdomen*** (Fig. 2A, B): tergites 1–8 dark brown with short brownish yellow setae; sternites 1–8 yellow with short yellow setae.

***Male terminalia*** (Figs 2A, 3C–E) brownish yellow with long brownish yellow setae. Ninth tergite short, middle margin with thick long setae, posterior margin slighltly concave; gonocoxite cylindrical, base of gonocoxite wider, inner side of gonocoxite with thick long setae, end of gonocoxite narrow; clasper of gonostylus slender, terminal spine decurved; lobe of gonostylus short and stout, posterior-outer margin swollen with thick setae; interbase with two branches, outer-lateral branch short and small with spine-like apex, inner branch as long as aedeagus, slightly wider at base, middle to posterior part slender and rod-shaped; aedeagus elongated and rod-shaped, with a thick base that gradually tapers toward the tip.

**Female** (*n* = 17): body length 6.6–7.8 mm, wing length 6.7–8.4 mm, antenna length 0.9–1.3 mm.

Female (Figs 2C, 3B) generally resembling male.

***Ovipositor*** (Figs 2C, 3B) brownish yellow; long, more than 1/4 as long as abdomen; cercus yellow, middle parts brown; cercus more than two times as long as tenth tergite, weakly upcurved on distal part; hypogynial valve yellow, middle parts brownish; hypogynial valve 1.2 times as long as eighth sternite, almost straight, tip ending at near level of middle of cercus.

##### Distribution.

China: Shaanxi (Xi’ an, Xianyang).

##### Etymology.

The species is named after Xi’ an Chanba National Wetland Park, where the type locality is located.

##### Remarks.

This new species is very similar to P. (P.) inconcussa in having similar wing venation (Fig. 5A) but can be separated from it by the aedeagus straight (Fig. 3C–E). In P. (P.) inconcussa, the aedeagus is apically directed dorsally (Fig. 5C–E). This new species is very similar to P. (P.) seticostata in having similar wing venation (Alexander 1936: pl. 1, fig. 10) but can be separated from it by the pronotum brownish yellow; prescutum brownish yellow with four dark brown stripes, middle two dark brown stripes longer, near pronotum darker; anepisternum brownish black; metapleuron light yellowish with pale pruinosity (Fig. 2A, B). In P. (P.) seticostata, the pronotum and mesonotum is almost uniformly dark brown; the pleuron is a little pale (Alexander 1936: 128).

#### Pseudolimnophila (Pseudolimnophila) inconcussa

Taxon classificationAnimaliaDipteraLimoniidae

(Alexander, 1913)

5E3BBCA1-4CE0-5345-A481-C9CB4FD53EED

Figs 1, 4, 5

Limnophila inconcussa Alexander, 1913c: 313 (description): Alexander 1913a: 209 (figure wing); Alexander 1913b: 295 (figure hypopygium); Alexander and McAtee 1920: 435 (figure wing).
Pseudolimnophila (Pseudolimnophila) inconcussa : Alexander, 1919: 917, 919; Alexander and Alexander 1973: 167; Nakamura 2005: 700; Nakamura 2018: 819, 820; Suguro and Kato 2023: 33, 34; Kato and Hiraga 2024: 31, 35; Kato and Kawase 2024: 7, 14.

##### Type species.

*Limnophila inconcussa* Alexander, 1913c. Type locality: Japan, Tokyo.

##### Specimens examined.

• 25 males, 4 females (CAU), China: Shaanxi, Yan’an, Ganquan county, Sigou village, 36.20N, 109.13E, 1173 m, 2024.VII.14, Bing Zhang and Yalian Wang, CAU: Limn: 129098, CAU: Limn: 129099, CAU: Limn: 129100, CAU: Limn: 129101, CAU: Limn: 129102, CAU: Limn: 129103, CAU: Limn: 129104, CAU: Limn: 129105, CAU: Limn: 129106, CAU: Limn: 129107, CAU: Limn: 129108, CAU: Limn: 129109, CAU: Limn: 129110, CAU: Limn: 129111, CAU: Limn: 129112, CAU: Limn: 129113, CAU: Limn: 129114, CAU: Limn: 129115, CAU: Limn: 129116, CAU: Limn: 129117, CAU: Limn: 129118, CAU: Limn: 129119, CAU: Limn: 129120, CAU: Limn: 129121, CAU: Limn: 129122, CAU: Limn: 129123, CAU: Limn: 129124, CAU: Limn: 129125, CAU: Limn: 129126; • 1 female (CAU), China: Shaanxi, Xianyang, Chunhua county, Dongzui village, 34.84N, 108.49E, 896 m, 2025.IX.13, Bing Zhang, CAU: Limn: 129127; • 10 males, 16 females (CAU), China: Shaanxi, Xianyang, Chunhua county, Crocodile Dam Scenic Area, 34.87N, 108.43E, 871 m, 2025.IX.13, Bing Zhang, Linjiang Zhu and Wen Ma, CAU: Limn: 129128, CAU: Limn: 129129, CAU: Limn: 129130, CAU: Limn: 129131, CAU: Limn: 129132, CAU: Limn: 129133, CAU: Limn: 129134, CAU: Limn: 129135, CAU: Limn: 129136, CAU: Limn: 129137, CAU: Limn: 129138, CAU: Limn: 129139, CAU: Limn: 129140, CAU: Limn: 129141, CAU: Limn: 129142, CAU: Limn: 129143, CAU: Limn: 129144, CAU: Limn: 129145, CAU: Limn: 129146, CAU: Limn: 129147, CAU: Limn: 129148, CAU: Limn: 129149, CAU: Limn: 129150 CAU: Limn: 129151, CAU: Limn: 129152, CAU: Limn: 129153; • 3 females (CAU), China: Shaanxi, Tongchuan, Yaozhou District, Zhaojin town, Anzi village, 35.11N, 108.71E, 1212 m, 2025.IX.13, Bing Zhang, Linjiang Zhu and Wen Ma (light trap), CAU: Limn: 129154, CAU: Limn: 129155, CAU: Limn: 129156.

##### Diagnosis.

Prescutum greyish black with four black stripes; mesopleura greyish black with pale grey pruinosity; *R_2_* moderately oblique, placed at fork of *R_3+4_*, approximately as long as 1/2 of *R_1_*; *M_1_* approximately as long as *M_1+2_*; abdominal sternites 1–8 greyish brown; interbase with two branches, outer-lateral branch short with spine-like apex, inner branch 3 times as long as outer-lateral branch, posterior part slender and rod-shaped; aedeagus a litter longer than interbase, apically directed dorsally.

##### Redescription.

**Male** (*n* = 35): body length 5.2**–**7.5 mm, wing length 6.7**–**7.6 mm, antenna length 1.3–1.8 mm.

**Figure 4.**
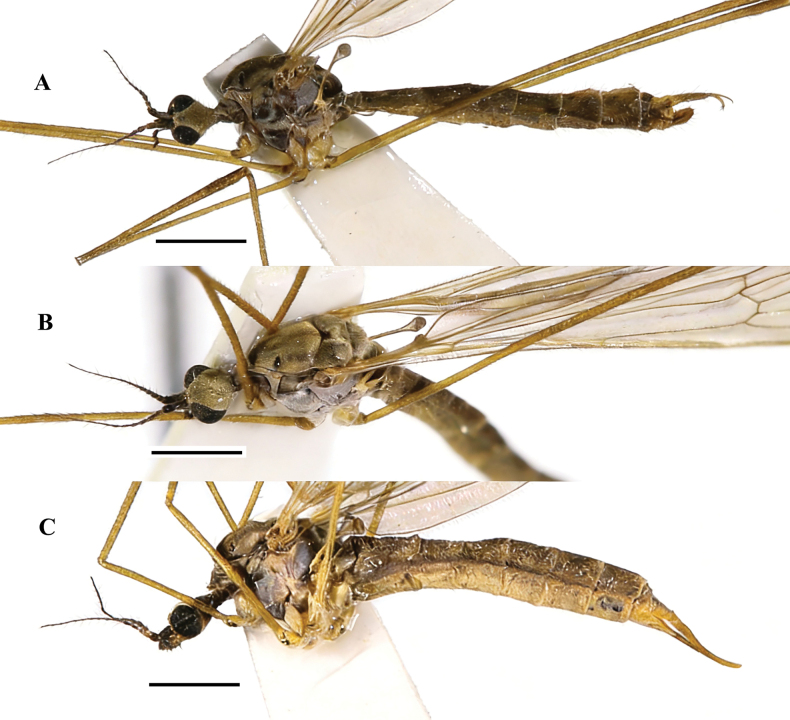
*Pseudolimnophila* (*P.*) *inconcussa*. **A**. Male habitus, lateral view; **B**. Male habitus, dorsal view; **C**. Female habitus, lateral view. Scale bars: 1.0 mm (**A**–**C**).

***Head*** (Fig. 4A, B) brownish grey with pale grey pruinosity. Vertex brownish grey with short setae; scape and pedicel brownish black; flagellomeres dark brown, cylindrical with longer verticils; rostrum and palpus greyish black with black setae.

**Figure 5.**
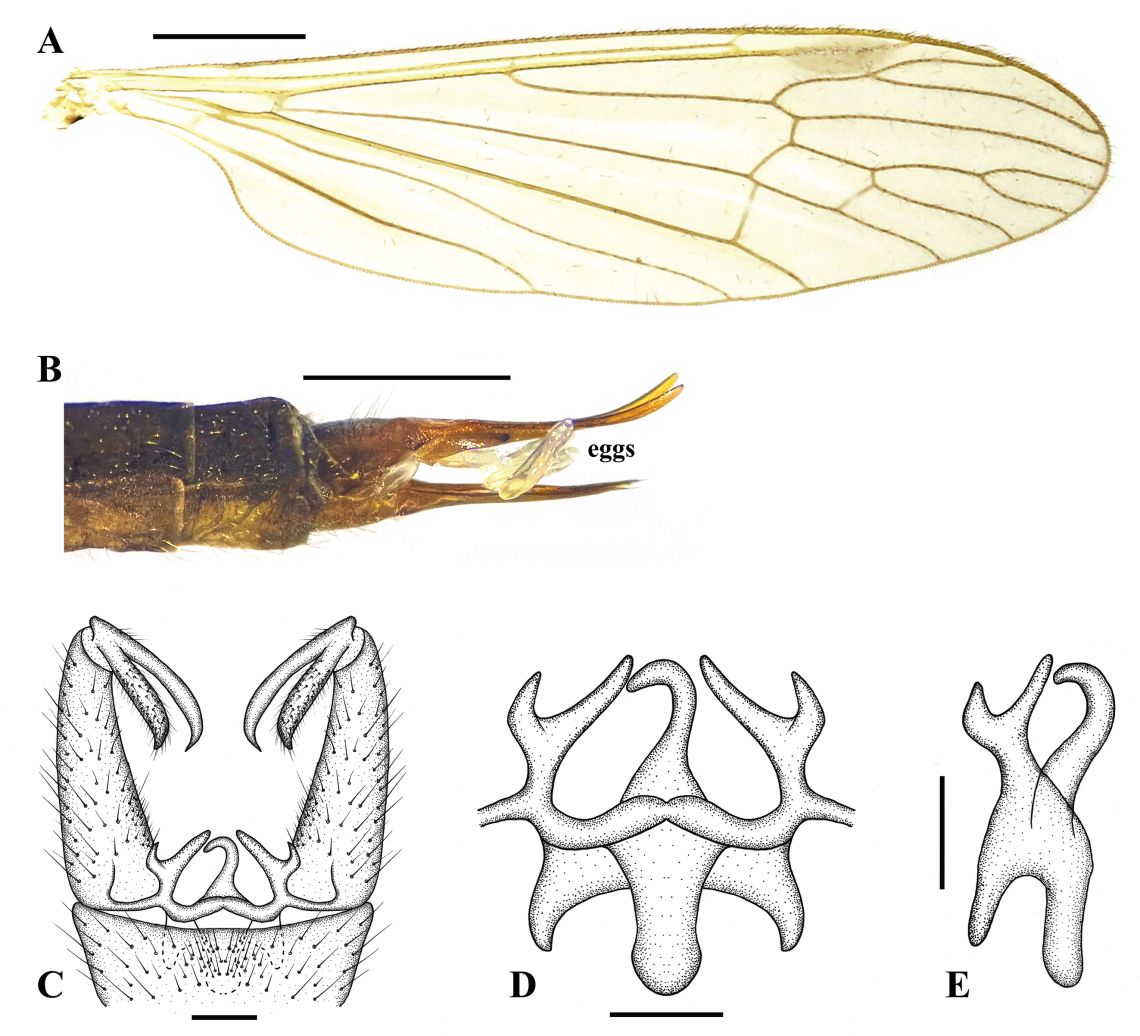
*Pseudolimnophila* (*P.*) *inconcussa*. **A**. Male wing; **B**. Ovipositor, lateral view; **C**. Terminalia, dorsal view; **D**. Aedeagal complex, dorsal view; **E**. Aedeagal complex, lateral view. Scale bars: 1.0 mm (**A**, **B**); 0.1 mm (**C**–**E**).

***Thorax*** (Fig. 4A, B) greyish black with pale grey pruinosity. Pronotum brownish grey; prescutum greyish black with four black stripes, middle two black stripes longer; prescutal suture black; scutum and scutellum greyish black; mesopleura greyish black with pale grey pruinosity, parts of near legs greyish yellow. Legs: coxa dark yellow with long yellowish setae; trochanter bright yellow; femur bright yellow with brown setae, posterior margin become brownish and thicker; tibia and tarsus brownish yellow with brown setae, posterior margin brown. Wing (Fig. 5A) pale greyish yellow hyaline, anal cell paler; pterostigma pale brown. Venation: *R_2_* moderately oblique, placed at fork of *R_3+4_*, approximately as long as 1/2 of *R_1_*; *M_1_* approximately as long as *M_1+2_*; *m-cu* not oblique, near beginning of cell *dm*. Halter (Fig. 4A, B) length approximately 1.1 mm, basal 1/5 of stem light yellow with pale pruinosity, remainder of stem and knob greyish.

***Abdomen*** (Fig. 4A, B): tergites 1–8 dark brown with short brownish yellow setae; sternites 1–8 greyish brown with short yellow setae.

***Male terminalia*** (Figs 4A, 5C–E) brownish yellow with long brownish yellow setae. Ninth tergite short, middle margin with thick long setae, posterior margin slighltly concave; gonocoxite cylindrical, base of gonocoxite wider, inner side of gonocoxite with thick setae, end of gonocoxite narrow; clasper of gonostylus slender, terminal spine decurved; lobe of gonostylus short and stout, posterior-outer margin swollen with thick setae; interbase with two branches, outer-lateral branch short with spine-like apex, inner branch 3 times as long as outer-lateral branch, posterior part slender and rod-shaped; aedeagus a litter longer than interbase, apically directed dorsally.

**Female** (*n* = 24): body length 6.4–7.8 mm, wing length 6.9–8.6 mm, antenna length 1.5–1.6 mm.

Female (Figs 4C, 5B) generally resembling male.

***Ovipositor*** (Figs 4C, 5B) brownish yellow; long, 1/3 as long as abdomen; cercus brownish yellow; cercus three times as long as tenth tergite, upcurved on distal part; hypogynial valve brownish; hypogynial valve more than two times as long as eighth sternite, almost straight, tip ending at near level of 3/4 of cercus.

##### Distribution.

China: Shaanxi (Yan’ an, Xianyang, Tongchuan), Guangdong, Guangxi, Taiwan; Russia: Primorskiy kray, Sakhalin (Kuril Is); South Korea; Japan (Hokkaido, Honshu, Shikoku, Kyushu).

##### Remarks.

This species is reported for the first time from Shaanxi Province.

#### Pseudolimnophila (Pseudolimnophila) shanxiensis

Taxon classificationAnimaliaDipteraLimoniidae

Shui, Zhang B. & Yang sp. nov.

F4BAB931-00F7-5ACD-B619-78307BBD8034

https://zoobank.org/F427D003-74E3-4DBA-8784-28FFCAF87E4D

Figs 6, 7

##### Type material.

***Holotype***: • male (CAU), China: Shanxi, Jinzhong, Lingshi county, Shigaoshan Mountain Scenic Area, 36.73N, 111.96E, 1416 m, 2024.VII.19, Bing Zhang and Yalian Wang, CAU: Limn: 129157. ***Paratypes***: • 1 male, 7 females (CAU), China: Shanxi, Jinzhong, Lingshi county, Shigaoshan Mountain Scenic Area, 36.73N, 111.96E, 1416 m, 2024.VII.19, Bing Zhang and Yalian Wang, CAU: Limn: 129158, CAU: Limn: 129159, CAU: Limn: 129160, CAU: Limn: 129161, CAU: Limn: 129162, CAU: Limn: 129163, CAU: Limn: 129164, CAU: Limn: 129165; • 1 female (CAU), China: Shanxi, Linfen, Guxian county, Huoshan Mountain Provincial Nature Reserve, 36.46N, 111.88E, 1569 m, 2024.VII.20, Bing Zhang, CAU: Limn: 129166.

##### Diagnosis.

Prescutum brownish black with yellowish pruinosity; mesopleura brownish black with pale grey pruinosity; *R_2_* not oblique, placed after fork of *R_3+4_*, shorter than *R_2+3_*, approximately as long as 1/2 of *R_2+3+4_*; *M_1_* longer, approximately 2 times as long as *M_1+2_*; abdominal sternites 1–8 light yellow; interbase with two rod-shaped branches, outer-lateral branch a little shorter than inner branch, apex stubby; aedeagus short, as long as inner branch of interbase.

##### Description.

**Male** (*n* = 2): body length 6.4**–**6.5 mm, wing length 7.3**–**7.5 mm, antenna length 1.6–1.7 mm.

**Figure 6.**
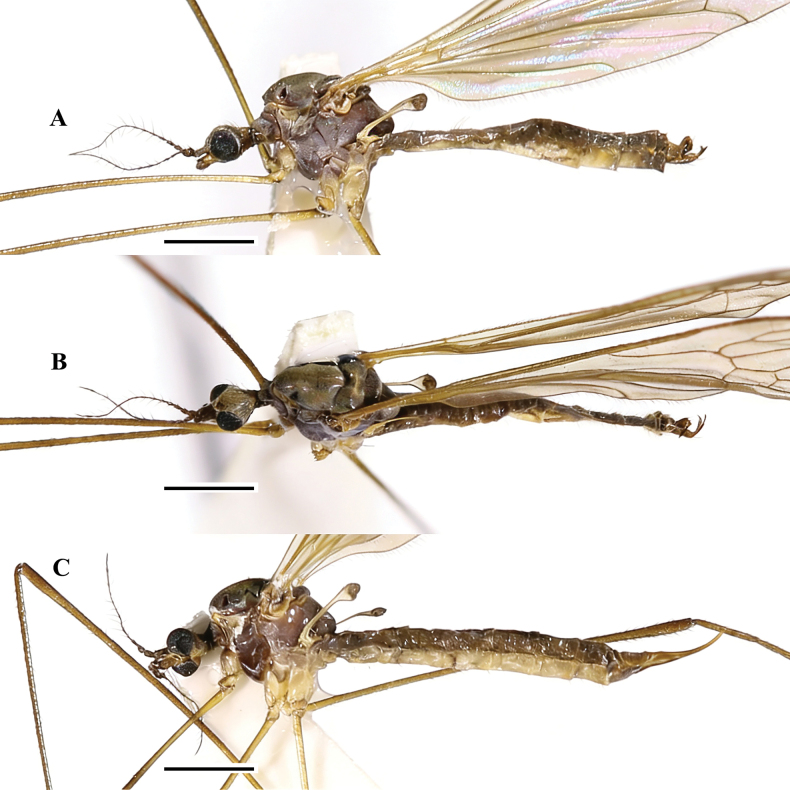
Pseudolimnophila (P.) shanxiensis Shui, Zhang B. & Yang, sp. nov. **A**. Male habitus, lateral view; **B**. Male habitus, dorsal view; **C**. Female habitus, lateral view. Scale bars: 1.0 mm (**A**–**C**).

***Head*** (Fig. 6A, B) brownish black with pale grey pruinosity. Vertex dark brown with short setae and yellowish pruinosity; scape and pedicel greyish black; flagellomeres cylindrical with longer verticils; rostrum and palpus brownish black with black setae.

**Figure 7.**
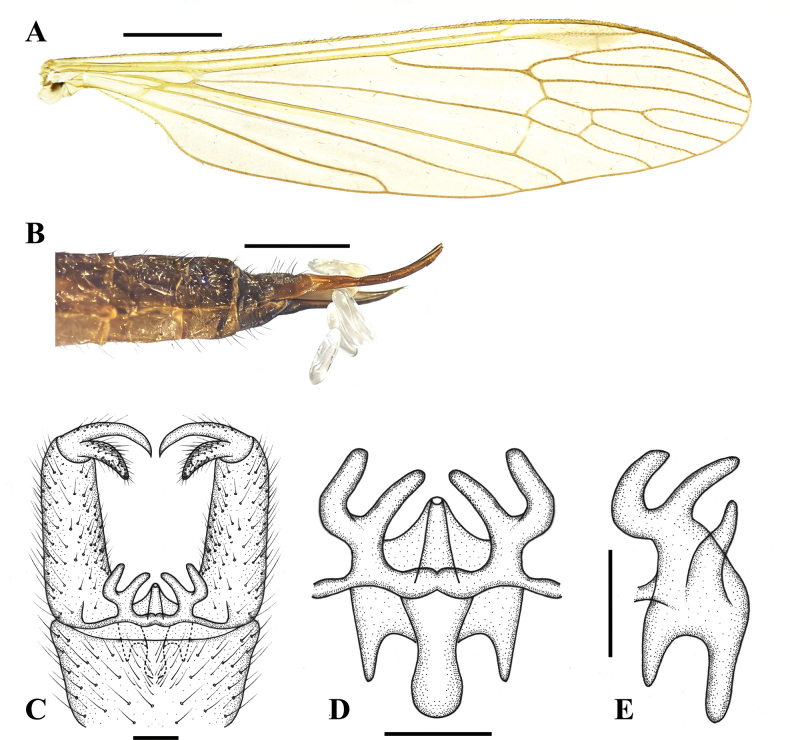
Pseudolimnophila (P.) shanxiensis Shui, Zhang B. & Yang, sp. nov. **A**. Male wing; **B**. Ovipositor, lateral view; **C**. Terminalia, dorsal view; **D**. Aedeagal complex, dorsal view; **E**. Aedeagal complex, lateral view. Scale bars: 1.0 mm (**A**, **B**); 0.1 mm (**C**–**E**).

***Thorax*** (Fig. 6A, B) brownish black. Pronotum dark brown; prescutum brownish black with yellowish pruinosity; prescutal suture black; scutum and scutellum brownish black; mesopleura brownish black with pale grey pruinosity, parts of near legs brownish. Legs: coxa bright yellow with long yellowish setae; trochanter brownish yellow; femur brownish yellow with brown setae, posterior margin become brown and thicker; tibia and tarsus brown with dark brown setae, posterior margin brownish black. Wing (Fig. 7A) pale greyish yellow hyaline, anal cell paler; pterostigma pale brown. Venation: *R_2_* not oblique, placed after fork of *R_3+4_*, shorter than *R_2+3_*, approximately as long as 1/2 of *R_2+3+4_*; *M_1_* longer, approximately 2 times as long as *M_1+2_*; *m-cu* moderately oblique, near base of cell *dm*. Halter (Fig. 6A, B) length approximately 1.2 mm, basal 1/2 of stem bright yellow, remainder of stem and knob greyish brown with pale pruinosity.

***Abdomen*** (Fig. 6A, B): tergites 1–8 dark brown with short brownish yellow setae; sternites 1–8 light yellow with short yellow setae.

***Male terminalia*** (Figs 6A, 6B, 7C–E) brownish black with long brown setae. Ninth tergite short, middle margin with thick long setae, posterior margin slighltly concave; gonocoxite cylindrical with thick setae; clasper of gonostylus slender, terminal spine decurved; lobe of gonostylus short and stout, posterior-outer margin swollen with thick setae; interbase with two rod-shaped branches, outer-lateral branch a little shorter than inner branch, apex stubby; aedeagus short, as long as inner branch of interbase.

**Female** (*n* = 8): body length 5.8–7.4 mm, wing length 6.9–7.6 mm, antenna length 1.4–1.6 mm.

Female (Figs 6C, 7B) generally resembling male.

***Ovipositor*** (Figs 6C, 7B) dark brown; long, more than 1/3 as long as abdomen; cercus brown; cercus three times as long as tenth tergite, upcurved on distal part; hypogynial valve dark brown; hypogynial valve more than two times as long as eighth sternite, weakly upcurved on distal part, tip ending at near level of 2/3 of cercus.

##### Distribution.

China: Shanxi (Jinzhong, Linfen).

##### Etymology.

This species is named after Shanxi Province, where its type locality is situated.

##### Remarks.

This new species is very similar to P. (P.) brunneinota in having similar wing venation (Alexander 1933: pl. 1, fig. 6) but can be separated from it by the interbase with two rod-shaped branches and stubby apex (Fig. 7C–E). In P. (P.) brunneinota, the interbase apex is spine-like (Alexander 1933: pl. 2, fig. 30). This new species is very similar to P. (P.) chikurina in having similar wing venation (Alexander 1930: pl. 1, fig. 8) but can be separated from it by the pronotum dark brown; prescutum brownish black with yellowish pruinosity, without stripe; mesopleura brownish black with pale grey pruinosity (Fig. 6A, B). In P. (P.) chikurina, the pronotum is brownish grey; the prescutum is brownish grey, median stripe is darker brown, the lateral stripes less distinct; the pleuron is brownish grey (Alexander 1930: 70).

## Supplementary Material

XML Treatment for Pseudolimnophila (Pseudolimnophila) chanbaensis

XML Treatment for Pseudolimnophila (Pseudolimnophila) inconcussa

XML Treatment for Pseudolimnophila (Pseudolimnophila) shanxiensis

## Additional information

### Use of AI

No use of AI was reported.

### Author ORCIDs

Bing Zhang https://orcid.org/0000-0002-3996-1760

Ding Yang https://orcid.org/0000-0002-7685-3478

### Data availability

All of the data that support the findings of this study are available in the main text.
